# Supplementary material for: Are carbon emissions trading and green financial instruments synergistic? -Comprehensive quantitative research based on content analysis
Source: PLoS One. 2024 Mar 7;19(3):e0298601. doi: 10.1371/journal.pone.0298601 (PMC10919601; doi:10.1371/journal.pone.0298601)
Supplement: S1 Data — (ZIP) [file pone.0298601.s001.zip › Data/地级市Z_F工作报告.zip!/░▓╗╒/═¡┴Ω╩╨/└┤╫╘╣1⁄2╓┌║┼í╛┬φ┐╦╩2╛▌═°í┐_2014.docx]

各位代表：

    现在，我代表市人民政府，向大会报告工作，请予审议，并请市政协委员和其他列席人员提出意见和建议。

    一、2013年工作回顾

    刚刚过去的2013年，宏观经济形势复杂多变，转型发展压力与日俱增。在省委、省政府和中共铜陵市委的坚强领导下，全市人民认真贯彻落实党中央决策部署，努力稳增长、调结构、促改革、惠民生，基本完成了年度目标任务【1】，经济社会发展稳中有进、稳中向好。预计，全年实现地区生产总值680亿元，增长11.5%。财政收入130.1亿元，其中地方收入64.2亿元，同口径增长4.2%。全社会固定资产投资650亿元，增长21.5%。社会消费品零售总额156亿元，增长13.5%。进出口总额56亿美元，增长57%。城镇居民人均可支配收入27154元、农民人均纯收入11226元，分别增长10%和14%。居民消费价格上涨1.9%。节能减排指标全面完成。

    一年来，我们聚焦铜陵转型发展中的突出问题、群众关注的热点问题，扎实开展项目推进年活动，组织实施土地房屋征收拆违、政策性住房清查等专项行动，形成了真抓实干、合力攻坚的良好局面。我们深入开展文明创建活动，创新社会治理，城市文明程度指数测评居全国地级城市第12名，首捧全国社会管理综合治理最高奖“长安杯”。我们紧紧围绕生态环境建设，强力开展石料矿山和选矿行业专项整治，大力推进美好乡村建设、森林增长和绿化提升工程，城乡面貌明显改善。我们积极抢抓政策机遇，成为国家节能减排财政政策综合示范市、循环经济示范创建市等16项新的试点示范，为转型发展增加了新的支点。

    第一，保持经济平稳较快增长。及时出台促进经济持续健康发展31项举措，大力实施民营经济促进计划、中小企业希望工程【2】，启动工业企业技改三年行动计划【3】，争取上级专项转移支付资金30.8亿元，兑现扶持企业资金3.5亿元、减免企业税费5.8亿元，净增规模以上企业67户，均创历年之最。着力推进重点项目建设，新开工亿元以上项目120个、竣工80个；实施亿元以上战略性新兴产业项目80个、服务业项目75个。全年，铜加工材突破100万吨，战略性新兴产业产值增长20%，实现了稳增长和调结构的有机统一。

    第二，强化转型发展平台建设。制订了推动开发园区转型发展的政策措施。开源金属再生产业园建设取得重要进展。口岸核心能力建设通过国家验收。建成安徽铜基新材料产业技术创新战略联盟，铜产业技术标准服务平台投入使用。中科院皖江新兴产业技术发展中心引进创新团队6个。主持或参与起草14个国家行业标准、占全省25%以上，新增国家高新技术企业20家，六国化工技术中心成为国家级中心。国家农业科技园区申报成功，国家现代农业示范市建设扎实推进。

    第三，提高城乡建设管理水平。基本完成新一轮城市总体规划修编，完成城市绿道系统、湖链城市等9个推动城市转型的专项规划编制。

    京福高铁铜陵长江公铁大桥顺利合龙，铜南宣高速铜陵段全面复工，东大路、沿江公路一期建成通车，新增一级公路60公里。新建和改造主城区道路49.2公里。基本实现主城区干支管网雨污分流，完成长江西路等区域排水能力提升工程。启动国家“智慧城市”试点市建设。完成10个省级美好乡村重点示范村建设任务，犁桥村被评为“中国美丽宜居村庄”。

    第四，加大城乡环境整治力度。实施节能减排重点示范项目64个。稳步推进横港扫把沟区域环境综合整治，狼尾湖治理主体工程完工，滨江生态岸线进入亲水观光带建设阶段。关闭石料矿山21个、小码头9个，取缔小选矿厂115个，超限超载和扬尘污染专项整治成效明显，城区可吸入颗粒物日均浓度下降2%。加大生态修复力度，造林面积2.1万亩，建成森林长廊示范段31公里、城市绿色廊道18.7公里，完成600亩废弃矿山生态修复。

    第五，深化重点领域改革创新。成立了市土地管理委员会，组建了新建投公司、市公共资源交易监督管理局。完成县级公立医院改革、港航地方海事管理体制改革。城乡户籍实现一元管理，城乡居民医保并轨运行。开展城市社区居委会“海选”试点、全面实行直选，村民理事会在美好乡村建设中发挥了积极作用。建成了市级社会组织培育中心。扩大了政府购买公共服务试点。深入开展行政审批事项第五轮清理，开通网上审批服务，成为全省行政审批事项最少的城市。

    第六，不断提升民生保障水平。高质量实施民生工程，累计投入资金13亿元，城乡居民受益面95%以上。企业退休人员养老金及时调标，被征地农民基本养老金标准提高13%以上；职工医保实现市级统筹，在全省率先开展大病医疗保险和异地就医联网结算，社会保障“一卡通”【4】顺利实施。城乡低保、农村五保对象集中供养标准提高11.3%，农村低保人均补差居全省首位。改造了一批养老服务机构，新增老年活动室36个。新开工保障性安居工程2万多套、竣工1.89万套，改造农村危房2600户。

    第七，深入推进和谐社会建设。“中国好人”当选比例全国领先。市辖区全部通过国家义务教育均衡发展验收认定。市及所有县区再获“国家科技进步考核先进”。成为全省首批知识产权示范市。人民医院门诊楼等7个卫生项目建成使用，新增三甲专科医院2所。乡镇综合文化站和农家书屋覆盖率100%，城市社区文化项目、农村文化墙项目在全国获奖。成功举办第十一届运动会暨第三届全民健身运动会。连续7次获得省双拥模范城称号。妇女儿童、民族宗教、外事侨务、对台、档案、史志、新闻出版、地震、气象等事业取得新进步。法治城市创建、社会治安综合治理成为全国先进。安全生产形势总体平稳。

    第八，切实加强政府自身管理。完善政府工作规则，改进机关作风、密切联系群众。扎实开展整治“庸懒散奢”、机关办公用房清查等活动。行政审批办理时限压缩到5个工作日内。严格支出管理，“三公”经费【5】支出下降16.2%。自觉接受市人大法律监督、工作监督和政协民主监督，按时办结人大代表建议183件、政协提案271件。推进廉政风险防控标准化建设，强化行政监察和审计监督。整合政府服务热线，基本实现“一个号码找政府”。深化政务公开工作，建立群众代表座谈会制度。

    各位代表！这些成绩的取得，凝聚了全市人民的智慧、心血和汗水，是省委、省政府和市委正确领导的结果。在此，我代表市人民政府，向全市人民，向人大代表、政协委员，向各民主党派、工商联、无党派人士和各人民团体，向驻铜部队和单位，向所有关心、支持、参与铜陵建设发展的同志们、朋友们，表示衷心的感谢，并致以崇高的敬意！

    我们清醒地认识到，铜陵发展中还存在不少困难和严峻挑战。资源型城市的困局仍未打破，土地、环境约束日益加剧，转型发展任务依然艰巨。政府职能转变远未到位，一些工作人员依法履职能力有待提高，“四风”问题仍很突出，腐败现象在一些领域多发高发。对此，我们必须高度重视，切实加以解决。

    二、2014年政府工作安排

    当前，我国经济进入增长速度换挡期、结构调整阵痛期、扩张政策消化期，外部环境不确定因素较多。同时，全面深化改革的红利、长江经济带开发战略的实施等，将为我市发展注入新的活力和动力。按照市委九届十一次全会的部署，今年政府工作的总体思路是：坚持以稳中求进为工作总基调，以全面深化改革为根本动力，以加快转型发展为基本出发点，以提高发展质量为工作着力点，促进经济平稳发展，确保社会和谐稳定。

    经济社会发展的主要预期目标是：全市生产总值增长10%以上；财政收入增长10%左右；全社会固定资产投资增长18%以上；社会消费品零售总额增长14%左右；城乡居民人均可支配收入与经济发展同步；新增就业2.2万人；居民消费价格涨幅控制在3.5%左右；确保完成国家下达的节能减排年度目标任务。

    重点抓好以下六个方面工作：

    （一）提高投资质量效益

    开展重大项目提质提效年活动。围绕有利于调整结构、改善环境、增加收入和保障民生，深入谋划、强力推进项目工作，持续扩大有效投入。继续推进土地房屋征收拆违专项行动，简化优化项目审批流程，促进项目加快落地和竣工投产。紧盯六大战略性新兴产业，健全招商引资考核机制，充分发挥产业政策、各级财政专项资金的引导作用。全年计划建设亿元以上重点项目310个，完成投资468亿元，增长20.6%。其中，新开工亿元以上项目107个、竣工81个。全力抓好抓实五类重点项目，其中调结构项目120个、节能减排项目134个、交通城建项目25个、现代服务业项目50个、民生和社会事业项目14个。

    加强政府性投资和融资管理。量力而行、合理确定政府投资项目，全年安排各类城乡建设项目200个以上、投资200亿元。其中各级政府投资55亿元，市本级为40亿元，主要用于建设重大交通基础设施、改善城乡生态环境、解决居民住行困难、发展社会事业。加强政府投资项目管理，确保工程质量，降低工程造价，严格责任追究，努力提高投资效益。加强政府融资管理，控制债务规模，优化债务结构，降低融资成本，做到风险可控。创新城乡建设投融资机制，加快制订鼓励民间资本进入基础设施、公用事业和社会事业的具体办法，抓好在供排水领域的试点，逐步建立城乡建设投入产出的良性循环机制。

    积极创造社会投入的良好环境。保持去年稳增长31条措施不变，稳定企业负担水平。继续实施百亿元企业服务计划，鼓励大型骨干企业兼并重组、做大做强。深入实施民营经济促进计划、中小企业希望工程，力争过亿元民营企业突破60户，民营经济占比提升1.5个百分点。多渠道帮助企业解决融资难问题，完善扩大银行信贷投放的激励政策、风险控制措施，支持各类融资担保机构扩大贷款担保规模。主动做好企业上市、区域集优票据【6】发行等直接融资服务，扩大企业直接融资规模。综合运用贴息担保、银企对接等措施，确保小微企业贷款增速不低于全市平均水平、增量不低于上年同期水平。

    （二）加快产业转型升级

    构建现代产业体系。落实工业企业技改三年行动计划年度任务，实施节能减排财政政策综合示范项目，综合运用土地、环保、能耗等刚性约束的倒逼机制，加快传统产业改造升级，提升传统优势产业竞争力。实施电子信息、化工新材料、先进装备制造、光电产业等百亿元产业集群培育计划，推动新兴产业集聚发展。抢抓长江经济带开发机遇，着力推进有色金属国际物流园等项目，建成甩挂中心，努力发挥港口区位优势，建设全国新兴物流节点城市。支持铜官山区IT产业园、铜都金融中心和开发区数字传媒园区建设。大力发展健康养老和社区服务业，积极发展铜文化创意产业，加快融入皖南国际旅游文化示范区。

    推进转型平台建设。落实促进开发园区转型发展的各项政策，提高产业支撑能力，园区规模以上工业总产值突破1000亿元，财政收入突破50亿元。开发区完成投资不低于150亿元，新开工10亿元以上项目不少于2个。示范区完成投资不少于30亿元，新开工10亿元以上项目不少于3个。各省级开发区要重点发展2-3个产业集群。继续做好综合保税区的申报争取工作。争取铜期货交割仓库落户。加快国家“智慧城市”试点市建设。确保“圈区管理”【7】通过验收并投入使用。

    强化科技人才支撑。深化产学研合作，办好中科院皖江新兴产业技术发展中心等创新平台。争创省级高新技术开发区1家，组建省级以上研发机构3家，新增国家高新技术企业15家，培育壮大一批科技中小企业。加快发展铜陵国家电子基础材料及新型元件高新技术产业化基地，积极发挥铜基新材料产业技术创新战略联盟的作用，打造光电产业技术创新战略联盟。完善网上技术交易市场和仪器设备、产品检验开放共享平台。争创国家知识产权示范市。积极推动国家铜铅锌及制品质检中心二期建设。通过全国质量强市示范城市验收。加大人才培养和引进力度，建立人才、科研股权和分红激励机制，在引进高层次人才方面有新突破。

    （三）综合整治城乡环境

    强力推进节能减排。确保完成节能减排财政政策综合示范市、国家循环经济示范创建市年度工作任务，启动千亿铜产业链节能减排示范、工业园区热能梯级利用等重点项目，深入推进4个国家循环化改造园区建设。把大气污染防治作为政府一号民生工程来抓，强化工业废气、建筑运输扬尘、机动车尾气等治理，扩大石料矿山和选矿行业整治成果，依法淘汰小选矿厂，依法关闭违规排放的小化工、手续不全的小码头，全面完成火力发电脱硫脱硝设施改造，升级改造铜冶炼、钢铁（球团）、水泥企业以及燃煤锅炉的除尘设施。抓好交通、建筑等其他领域节能减排。

    开展三线三边【8】治理。以“治脏、治乱、治污、植绿”为内容，把拆违治乱与落实森林增长、绿化提升工程紧密结合起来，做到边拆违、边整治、边绿化，打造城市森林长廊和沿线风景带。全年新增造林绿化面积2.45万亩，建成森林长廊示范段20公里。完成绿化提升面积400万平方米，建设城市绿色廊道8.4公里，恢复矿山植被3000亩。落实横港扫把沟区域环境综合整治年度任务。加快天井湖、翠湖水体环境治理，启动大铜官山公园、东湖湿地公园建设，建成滨江生态岸线。

    严格生态环境监管。加强城乡生态用地保护，根据新一轮城市总体规划，在全市范围内划定永久性生态用地。健全环境功能分区、环境行为评价、损害赔偿、责任追究等制度，进一步提高环境准入门槛。提高环境执法能力，加大执法检查力度，始终保持高压态势，严厉打击违规排放行为。启动雾霾天气重度污染应急处置工作。强化城市集中式饮用水水源保护，适时启动饮用水源取水口搬迁工程。完成重金属污染治理年度计划。建成餐厨废弃物处理项目。

    （四）推进城乡一体发展

    深化城乡一体化综合改革。以提升城乡公共服务均等化水平为重点，完善一元化户籍制度改革配套政策，使城乡居民享有平等的公共服务和社会保障、社会救助，促进城乡共同发展。围绕增加农民财产性收入，积极推进农村产权制度改革。巩固和完善农村产权确权登记发证成果，加强农村土地流转管理，确保农民合法权益。探索农村集体建设用地、土地承包经营权、农民宅基地和住房的资产化改革，推进农村和农民资产的资本化试点，促进生产要素城乡流动。争取成为国家新型城镇化试点市、全国农村产权制度改革试点市。

    加强城乡规划建设管理。坚持全域规划理念，以新一轮城市总体规划为引领，加快各类专项规划编制工作，建立覆盖城乡、统一完备的城乡规划体系，有效指导城乡建设。探索试行“多规合一”，把经济社会发展规划、土地利用总体规划、城市总体规划有机统一起来，更加准确地界定新老城区、东南部城区、开发园区、山区、圩区（包括江心洲）的发展定位。强化土地用途管制，把耕地红线扩展到永久性基本农田、永久性生态用地。围绕做好“山、水、田、路、亮、绿”六篇文章，加强城乡建设管理，畅通城市道路内循环，打通城市外环线，新（续）建和改造城市道路185公里，完成沿江快速通道、朱永路、滨江大道北段二期等工程建设，新增公交车60台，再开通乡镇公交线路2条。新建、改建城市燃气管网63公里、供水管网51公里、污水管网55公里。严禁违规建设，整治市容市貌、村容村貌，提高城乡管理精细化水平。

    扎实推进美好乡村建设。建成省级中心村16个、市级中心村20个，着力串珠、连线、成片，建设田园化的美丽家园。争取铜陵县成为全国美丽乡村建设标准化试点。继续推进农村清洁工程、县乡公路升级改造和农村危房改造工程，实施渔民上岸安居工程，改善农村人居环境。加大国家现代农业示范区、农业科技示范园建设的推进力度。建设旱涝保收标准农田示范项目2万亩，建成“菜篮子”标准化基地2000亩，种植油用牡丹5000亩以上，打造铜陵牡丹、白姜加工工业区。加快发展家庭农场等农村新型经营主体，鼓励农民以承包经营权入股发展农业产业化经营。

    （五）着力保障基本民生

    精心实施民生工程。坚持既尽力而为又量力而行，务实安排民生工程项目。全年计划安排财政资金14亿元，实施38项民生工程，重点解决棚户区改造、大气污染治理、停车场建设管理等群众最关心的问题。加强项目绩效管理，创新项目实施、投入和管理机制，做到项目由群众提出、过程由群众参与、效果由群众评议，切实把民生工程办成群众满意的民心工程。

    加强社会保障工作。坚持把促进就业作为优先目标，创建充分就业城市。推进全民创业，继续实施千人创业扶持计划，争创国家级创业型城市。巩固城乡居民医保并轨成果，发挥大病保险保障功能。推进各类养老保险转移接续，扩大社会保险覆盖面。完善“1+6”新型城乡社会救助体系【9】，建立城乡低保、五保供养、重点优抚对象抚恤等保障标准自然增长机制。新开工保障性安居工程6000套、供应保障房1000套，健全保障房分配、管理、退出机制。改革完善住房公积金制度。

    提升公共服务水平。探索名校托管、设立分校等办学模式，扩大优质教育资源覆盖面，继续实施学前教育三年提升计划，深化职业教育产教结合。实施“强院名医”培养工程，开展市立医院、市中医院法人治理结构改革试点，组建5个医疗联合体，完善分级诊疗模式。加快现代公共文化服务体系建设，提升城乡社区文化水平，组建图书馆、博物馆、文化馆理事会。落实“单独二孩”政策，力争全市60%家庭达到幸福家庭标准。建设养老康复指导中心、市级养老服务信息平台，新建3所社区老年人日间照料中心，养老机构床位数增长15%。

    争创全国文明城市。广泛开展社会主义核心价值观教育，扎实开展育人育德、窗口行风、志愿服务等系列活动，继续深化“铜陵好人”评选等活动。完善全民健身机制，积极备战省第十三次运动会。办好第十三届中国（铜陵）青铜文化博览会。推动新闻出版、广播影视、文学艺术事业繁荣发展。做好国防动员、民兵预备役和人防工作，争创新一轮全国双拥模范城。发挥工会、共青团、妇联等人民团体作用，加强民族宗教、外事侨务、对台、气象、档案、史志、新闻出版、慈善、红十字、防震减灾等工作。

    （六）完善基层社会治理

    创新社会治理方式。深化社区综合体制改革，厘清社区的公共服务职能和居（村）委会的自治职能，指导组建小区业主委员会，健全民主协商机制。培育多元治理主体，改革社会组织管理，推进行业协会、商会与政府机关脱钩，积极发展各类志愿服务组织，支持各类主体平等参与社会治理。实施社区服务提升工程，打造一批城乡精品示范社区，搭建全市统一的社区公共服务综合信息平台。

    维护社会和谐稳定。坚持系统治理、依法治理、综合治理、源头治理，完善基层信访工作机制，加强矛盾纠纷排查化解，建立网上信访平台，进一步畅通和规范群众诉求表达、利益协调、权益保障渠道，切实维护群众合法权益。健全重大决策社会稳定风险评估机制，完善社会治安防控体系，依法惩治违法犯罪活动，提升人民群众的安全感、幸福感。健全安全生产责任体系，加强重点行业和领域安全隐患排查整治，坚决遏制重特大事故发生。强化食品药品安全监管，提高保障公共安全和应对突发事件能力。

    三、加强政府自身建设

    地方政府是国家治理体系的重要环节。必须按照党的十八届三中全会要求，大胆解放思想，推进自身改革，增强行政能力，完善治理结构，激发市场活力，促进社会和谐。

    正确履行政府职能。深化行政审批制度改革，减少和规范行政审批事项和条件，建立行政审批标准化管理流程，探索建立“负面清单”【10】制度，实施注册资本登记制度改革。开展财政管理改革创新年活动，启动全口径预算编制工作；严格财政支出管理，建立资金使用全过程动态监控机制；整合财政专项资金，改进分配机制，提高使用效益。健全公开透明、公平竞争的公共资源交易市场，所有公共资源交易活动必须在市场进行。深化规范行政执法示范市建设，规范行使行政执法职能，完善执法程序，严肃追究“不作为”责任，严肃惩处滥用执法权行为。抓好行政复议规范化建设，及时纠正违法和不当行政行为。按照中央和省统一部署，积极稳妥推进政府机构改革。加快市县工商质监食品药品监管体制改革。建立事业单位法人治理结构，推进事转企或社会组织。逐步扩大政府购买公共服务的范围。

    拓宽监督制约渠道。逐步推行政府工作部门权力清单制度，完善绩效考核评价体系。整合政府信息资源，建设统一的政务信息平台，扩大政务信息公开范围，做到决策公开、管理公开、服务公开、结果公开，全方位接受社会监督，让权力在阳光下运行。提升“12345”市长热线、行风热线功能，办好政府微博、市民论坛，建设“网上政府”，畅通民意表达途径，打造服务群众“直通车”。切实发挥政府内部监督的作用，加强行政监察，坚决治理“官场病”；加大审计监督力度，强化审计成果运用。自觉接受人大法律监督、工作监督和政协民主监督，完善向市人大报告和向市政协通报工作制度，认真办理人大代表建议和政协提案，充分听取各民主党派、工商联、无党派人士和各人民团体意见。

    切实提高行政效能。认真开展以“为民、务实、清廉”为主题的党的群众路线教育实践活动，从群众最不满意的地方改起，从群众普遍期盼的事情做起，从群众长期受益的源头抓起。坚持实干兴铜，始终保持奋发有为的精神状态，多到基层接地气，多到一线解难题，加强督查督办，严肃行政问责，注重绩效评估，促进工作落实。认真落实党风廉政建设责任制和廉政准则，着力推进反腐倡廉制度建设，健全廉政风险防控机制。严格执行中央八项规定和省、市委规定，厉行节约、反对浪费，改革公务接待制度，深化公务用车改革，进一步清理整改机关办公用房。高质量完成政策性住房清查专项行动。牢固树立过“紧日子”思想，确保财政供养人员只减不增，确保“三公”经费只减不增，把有限的资金更多地用在改善民生上。

    各位代表！铜陵正处在改革再出发、转型正当时的重要关口，任务艰巨，时不我待。让我们在中共铜陵市委的坚强领导下，高举中国特色社会主义伟大旗帜，紧随“中国梦”的时代大潮，解放思想，开拓进取，同心同德，真抓实干，为全面深化改革、推动转型发展、建设幸福铜陵而努力奋斗！
